# Dimethylamine as the key intermediate generated in situ from dimethylformamide (DMF) for the synthesis of thioamides

**DOI:** 10.3762/bjoc.11.187

**Published:** 2015-09-23

**Authors:** Weibing Liu, Cui Chen, Hailing Liu

**Affiliations:** 1 College of Chemical Engineering, Guangdong University of Petrochemical Technology, 2 Guandu Road, Maoming 525000, P. R. China, Fax: +86-668-2923575; Tel: +86-668-2923956; 2 College Analytical and Testing Centre, Beijing Normal University, No. 19, Xinjiekouwai St., Haidian District, Beijing 100875, P. R. China; Tel: +86-15010928428

**Keywords:** aldehydes, dimethylformamide (DMF), elemental sulfur, ketones, thioamides

## Abstract

An improved and efficient method for the synthesis of thioamides is presented. For this transformation, dimethylamine as the key intermediate is generated in situ from dimethylformamide (DMF). All the tested substrates produced the desired products with excellent isolated yields.

## Introduction

Thioamides, a well-known structural element of many sulfur-containing molecules, synthetic agents [1–2], heterocycles, natural products and pharmaceuticals [3–8], have attracted considerable attention for their construction and use in organic synthesis [9–10]. Many compounds containing a thioamide motif are of medicinal significance and exhibit potent biological activities. These include for example opioid activity [11], immunosuppressive activity and DHODH inhibitory properties [12], activity against parasitic nematodes [13], and antituberculotic activity [14].

**Scheme 1:**
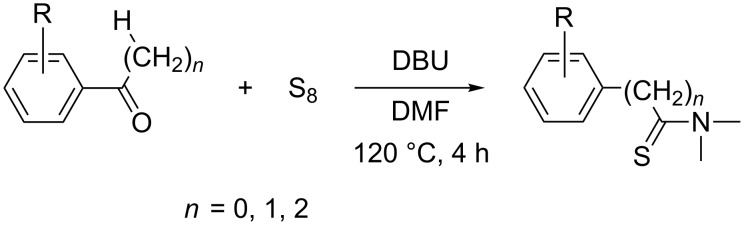
Synthesis of thioamide derivatives.

Consequently, a number of synthetic methods for the construction of this important unit have been established over the past decades [15–21]. However, some of these methods have limited applications, because of harsh conditions, low yields or the need of noble-metal catalysts. Therefore the development of novel and efficient methods for the construction of the thioamide motif is highly desirable. To avoid the disadvantages of the traditional methods, our group has developed an improved synthetic procedure to construct thioamides (Scheme 1).

## Results and Discussion

Our initial efforts focused on the optimization of the reaction conditions by employing 4-methoxybenzaldehyde (**1a**) as a model reactant to interact with elemental sulfur and DMF (Table 1). The reaction is completed after 4 h at 120 °C, providing 4-methoxy-*N*,*N*-dimethylbenzothioamide (**2a**) with 64% yield by using sodium acetate (AcONa) as the base (Table 1, entry 2). With regard to the catalytic activity of different bases (Table 1, entries 4–8), 1,8-diazabicyclo[5.4.0]undec-7-ene (DBU) showed the highest activity and afforded the desired product with 96% yield (Table 1, entry 7). It is worth mentioning that this conversion does not take place in the absence of a base (Table 1, entry 9). Lowering the temperature from 120 °C to 100 °C decreased the yield considerably (Table 1, entry 10). When the reaction was performed at room temperature no product was obtained at all (Table 1, entry 11).

**Table 1:** Optimization studies.^a^

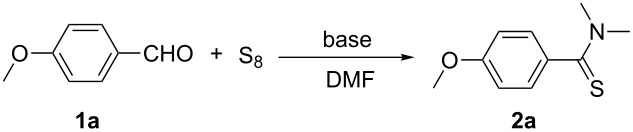				

Entry	Temp. ( °C)	Base (0.2 equiv)	Time (h)	Yield (%)^b^

1	120	AcONa	3	57
2	120	AcONa	4	64
3	120	AcONa	5	64
4	120	Na_2_CO_3_	4	25
5	120	KOH	4	15
6	120	DABCO	4	21
7	120	DBU	4	96
8	120	EtONa	4	17
9	120	none	4	none
10	100	DBU	4	79
11	rt	DBU	4	none

^a^Reaction conditions: **1a** (0.25 mmol), S_8_ (1.2 equiv, based on 1/8 S_8_), DMF (2.0 mL); ^b^GC yield.

Once the optimal conditions had been identified (Table 1, entry 7), next the scope of the reaction was investigated. Sixteen substrates including 12 substituted aldehydes and 4 ketones were screened and the results are presented in Table 2. As can be seen from the Table, all reactions proceeded smoothly and gave the corresponding thioamides **2a–p** exclusively in good to excellent isolated yields. It was also found that unsubstituted, mono-, di- and trisubstituted substrates regardless of the position (*o*-*, m*-*,* or *p*-) and the electronic properties of the substituents (electron-donating or –withdrawing) on the benzene ring were all compatible with the standard conditions. For example, methoxy-, *o*-, *m*-, and *p*-methyl-, ﬂuoro-, chloro-, or hydroxy-substituted aromatic aldehydes and ketones were all converted to their corresponding thioamides with excellent isolated yields. Finally, some aliphatic aldehydes such as phenylacetaldehyde (Table 2, entry 9), butyraldehyde (Table 2, entry 15) and pentanal (Table 2, entry 16) were subjected to the reaction. They were also found compatible with the standard conditions, and the corresponding products were isolated in moderate to good yields.

**Table 2:** Scope of the reaction.^a^

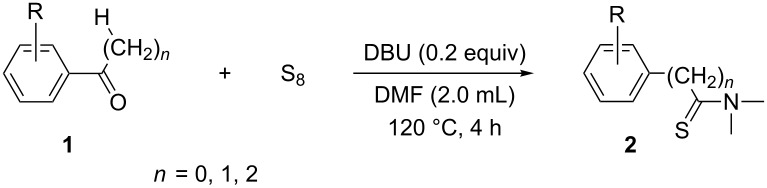			

Entry	Educt **1a**–**p**	Product **2a**–**p**	Yield (%)^b^

1	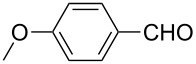 **1a**	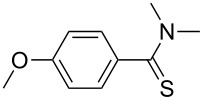 **2a**	90 (88)
2	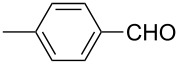 **1b**	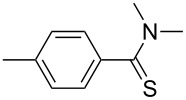 **2b**	84
3	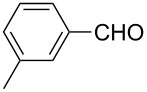 **1c**	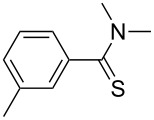 **2c**	82
4	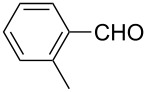 **1d**	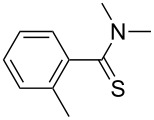 **2d**	82
5	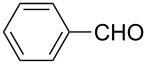 **1e**	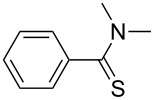 **2e**	83
6	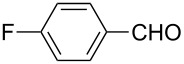 **1f**	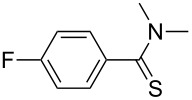 **2f**	80
7	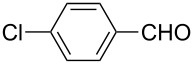 **1g**	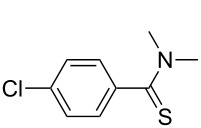 **2g**	80
8	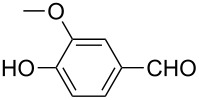 **1h**	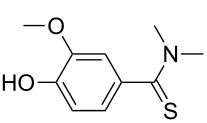 **2h**	89
9	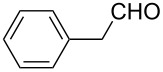 **1i**	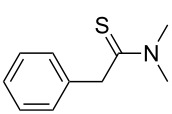 **2i**	83
10	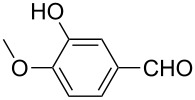 **1j**	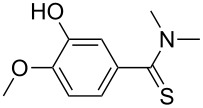 **2j**	88
11	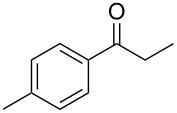 **1k**	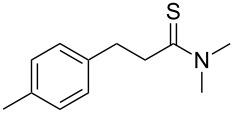 **2k**	80
12	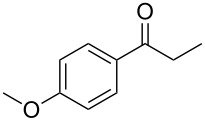 **1l**	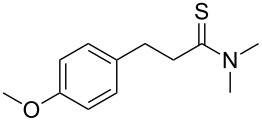 **2l**	84
13	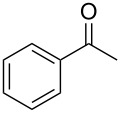 **1m**	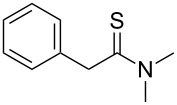 **2i**	77
14	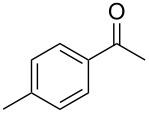 **1n**	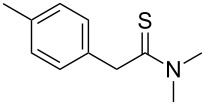 **2n**	79
15	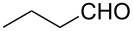 **1o**	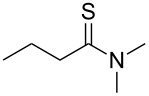 **2o**	63
16	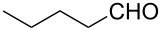 **1p**	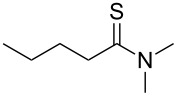 **2p**	67

^a^Reaction conditions: **1** (1.0 mmol), S_8_ (1.2 equiv, based on 1/8 S_8_); ^b^isolated yield. Number in parentheses is the isolated yield of **1** (10.0 mmol scale) after purification by column chromatography.

**Scheme 2:**
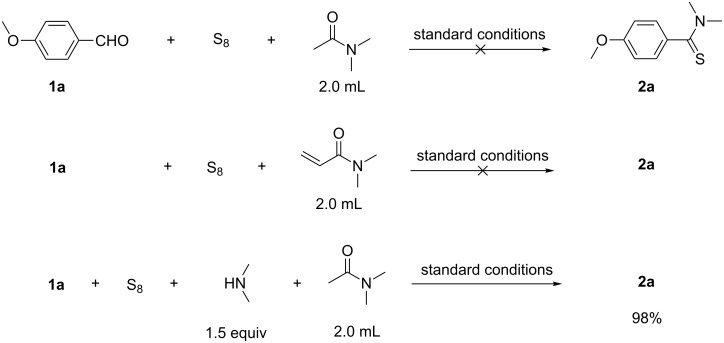
Control experiments.

In order to expand the scope and to get insight into the reaction mechanism of this protocol, three control experiments were conducted (Scheme 2). In the first two experiments the reaction was performed with *N*,*N*-dimethylacetamide (DMA) and *N*,*N*-dimethylacrylamide instead of DMF under the standard conditions. In both cases no product formation was observed, suggesting that neither DMA nor *N*,*N*-dimethylacrylamide is able to promote the reaction. However, repeating the reaction in DMA in the presence of *N*,*N*-dimethylamine the desired product **2a** was obtained in 98% yield. These results are proving the involvement of dimethylamine in this transformation and that it is in situ generated from *N*,*N*-dimethylformamide under the reaction conditions.

**Scheme 3:**
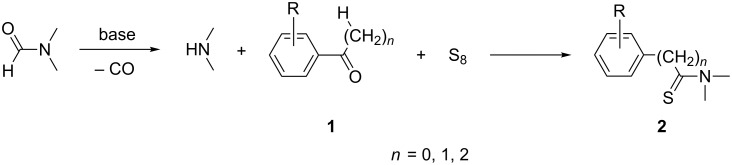
A mechanistic rationale for the synthesis of thioamides.

Based on the above experiments and the existing literature [22–24], a suggested mechanism is outlined in Scheme 3. The reaction starts with a base-induced cleavage of DMF to form the required dimethylamine according to the mechanism suggested by Van der Eycken, Hallberg and co-workers [25–26]. The subsequent step involves the classical Willgerodt–Kindler reaction as described by Amupitan and Darabi [27–28].

## Conclusion

In summary, an improved synthetic procedure for the synthesis of thioamides has been established. This protocol is applicable to a wide range of aldehydes and ketones yielding the thioamides with excellent isolated yields. For this transformation, DMF works not only as the solvent but also as the source of dimethylamine. The present method is more practical compared to the traditional strategies and complements the classical methods for the rapid construction of thioamides.

## Supporting Information

File 1 Full experimental details and copies of NMR spectra.
