# Supplementary material for: Dimethylamine as the key intermediate generated in situ from dimethylformamide (DMF) for the synthesis of thioamides
Source: Beilstein J Org Chem. 2015 Sep 23;11:1721–6. doi: 10.3762/bjoc.11.187 (PMC4660898; doi:10.3762/bjoc.11.187)
Supplement: File 1 — Full experimental details and copies of NMR spectra. [file Beilstein_J_Org_Chem-11-1721-s001.pdf]

Supporting information

for

Dimethylamine as the key intermediate generated in situ from dimethylformamide (DMF) for the synthesis of thioamides

Weibing Liu[1]*, Cui Chen[1] and Hailing Liu[2]*

Address: [1]College of Chemical Engineering, Guangdong University of Petrochemical Technology, 2 Guandu Road, Maoming 525000, P. R. China, Fax: +86-668-2923575; Tel: +86-668-2923956 and [2]College Analytical and Testing Centre, Beijing Normal University, No. 19, Xinjiekouwai St., Haidian District, Beijing 100875, P. R. China; Tel: +86-15010928428

*Corresponding author

Email: Weibing Liu - lwb409@aliyun.com; Hailing Liu - liuhailing@bnu.edu.cn

Full experimental details and copies of NMR spectra

General methods. All reactions were carried out at 120 °C for 4 h in a test tube equipped with a magnetic stirring bar. Solvents and reagents were purchased from Aldrich Chemicals or J & K Scientific Ltd. and were used as received. Petroleum ether (PE) refers to the fraction boiling in the 60–90 °C range. Thin-layer chromatography was performed using Qingdao-Haiyang 600 mesh silica gel plates (GF254), and samples were made visual with short wavelength UV light (254 nm). Melting points were measured on a melting point apparatus equipped with a thermometer and are uncorrected. IR spectra were recorded on a Bruker Vector 22 spectrometer as KBr pellets. ^{1}H NMR and ^{13}C NMR spectra were recorded on a 400 MHz spectrometer in $CDCl_3$ solutions with tetramethylsilane as the internal standard. Chemical shift (δ) values are given in ppm and coupling constants (*J*) in Hz. GC–MS spectra were obtained using an Agilent 6890N/5973 mass spectrometer and electron ionization (EI). HRMS was recorded on a commercial apparatus (ESI Source, TOF).

Typical procedure: 4-methoxy-*N*,*N*-dimethylbenzothioamide (2a, Table 2, entry 1). A mixture of 4-methoxybenzaldehyde (**1a**) (136 mg, 1.0 mmol), elemental sulfur (39 mg, 1.2 mmol), 1,8-diazabicyclo[5,4,0]undec-7-ene (DBU, 31mg, 0.2 mmol) and *N*,*N*-dimethylformamide (DMF, 2.0 mL) was added successively to the test tube and the resulting solution was stirred for 4 h at 120 °C. The mixture was then subjected to purification by preparative thin-layer chromatography (PE–EtOAc, 10:1) to afford product **2a**.

4-Methoxy-*N*,*N*-dimethylbenzothioamide (2a, Table 2, entry 1) [1]

^{1}H NMR ($CDCl_3$, 400 MHz) δ = 7.28 (d, *J* = 7.2 Hz, 2H), 6.85 (d, *J* = 7.2 Hz, 2H), 3.80 (s, 3H), 3.57 (s, 3H), 3.19 (s, 3H); ^{13}C NMR ($CDCl_3$, 100 MHz) δ = 201.2, 160.0, 135.8, 127.8, 113.4, 55.4, 44.3, 43.5.

***N*,*N*,4-trimethylbenzothioamide (2b, Table 2, entry 2) [1]**

^{1}H NMR ($CDCl_3$, 400 MHz) δ = 7.19 (d, *J* = 6.4 Hz, 2H), 7.13 (d, *J* = 6.4 Hz, 2H), 3.57 (s, 3H), 3.15 (s, 3H), 2.33 (s, 3H); ^{13}C NMR ($CDCl_3$, 100 MHz) δ = 201.5, 140.5, 138.6, 128.8, 125.8, 44.2, 43.3, 21.7.

***N*,*N*,3-Trimethylbenzothioamide (2c, Table 2, entry 3) [1]**

^{1}H NMR ($CDCl_3$, 400 MHz) δ = 7.21 (t, *J* = 6.4 Hz, 1H), 7.12 (d, *J* = 6.4 Hz, 1H), 7.11 (s, 1H), 7.05 (d, *J* = 6.4 Hz, 1H), 3.57 (s, 3H), 3.14 (s, 3H), 2.33 (s, 3H); ^{13}C NMR ($CDCl_3$, 100 MHz) δ =

201.5, 143.3, 138.1, 129.3, 128.2, 126.3, 122.6, 44.1, 43.1, 21.3.

***N*,*N*,2-Trimethylbenzothioamide(2d, Table 2, entry 4) [2]**

^{1}H NMR ($CDCl_3$, 400 MHz) δ = 7.65 (d, *J* = 7.6 Hz, 1H), 7.43 (t, *J* = 7.6 Hz, 1H), 7.27 (m, 2H), 3.55 (s, 3H), 3.28 (s, 3H), 2.68 (s, 3H); ^{13}C NMR ($CDCl_3$, 100 MHz) δ = 198.4, 141.8, 133.1, 132.6, 132.0, 131.8, 125.8, 42.4, 40.6, 21.9.

***N*,*N*-Dimethylbenzothioamide (2e, Table 2, entry 5) [1]**

^{1}H NMR ($CDCl_3$, 400 MHz) δ = 7.35 (m, 3H), 7.30 (d, *J* = 6.4 Hz, 2H), 3.60 (s, 3H), 3.16 (s, 3H); ^{13}C NMR ($CDCl_3$, 100 MHz) δ = 201.2, 143.4, 128.5, 128.3, 125.7, 44.1, 43.2.

4-Fluoro-*N*,*N*-dimethylbenzothioamide (2f, Table 2, entry 6)

Yellow crystals; mp 84-86 °C; IR ν_{max} (KBr): 1728, 1662, 1629, 1598, 1525, 1394, 1292, 1224, 1143, 835, 810 cm^{-1}; ^{1}H NMR ($CDCl_3$, 400 MHz) δ = 7.31 (t, *J* = 6.4 Hz, 2H), 7.04 (t, *J* = 8.0 Hz, 2H), 3.59 (s, 3H), 3.18 (s, 3H); ^{13}C NMR ($CDCl_3$, 100 MHz) δ = 200.2, 163.9, 161.4, 139.4, 128.0, 127.9, 115.4, 115.2, 44.2, 43.4; HRMS (ESI): calcd for $C_9H_{10}FNS^+$ [M+H$^+$] 184.0597, found 184.0592.

4-Chloro-*N*,*N*-dimethylbenzothioamide (2g, Table 2, entry 7) [3]

^{1}H NMR ($CDCl_3$, 400 MHz) δ = 7.32 (d, *J* = 7.6 Hz, 2H), 7.25 (t, *J* = 7.6 Hz, 2H), 3.59 (s, 3H), 3.17 (s, 3H); ^{13}C NMR ($CDCl_3$, 100 MHz) δ = 200.3, 141.6, 134.6, 128.5, 127.2, 44.1, 43.3.

4-Hydroxy-3-methoxy-*N*,*N*-dimethylbenzothioamide (2h, Table 2, entry 8) [4]

^{1}H NMR ($CDCl_3$, 400 MHz) δ = 6.92 (s, 1H), 6.81 (d, *J* = 8.0 Hz, 1H), 6.75 (d, *J* = 8.0 Hz, 1H), 5.90 (s, 1H), 3.85 (s, 3H), 3.55 (s, 3H), 3.18 (s, 3H); ^{13}C NMR ($CDCl_3$, 100 MHz) δ = 201.1, 146.3, 146.2, 135.3, 119.1, 113.8, 110.0, 56.0, 44.4, 43.5.

***N*,*N*-Dimethyl-2-phenylethanethioamide (2i, Table 2, entry 9) [1]**

Obtained starting from 2-phenylacetaldehyde (**1i**).^{1}H NMR ($CDCl_3$, 400 MHz) δ = 7.33 (m, 5H), 4.32 (s, 2H), 3.50 (s, 3H), 3.20 (s, 3H); ^{13}C NMR ($CDCl_3$, 100 MHz) δ = 200.6, 135.6, 128.8, 128.3, 128.0, 50.9, 44.1, 42.2.

3-Hydroxy-4-methoxy-*N*,*N*-dimethylbenzothioamide(2j, Table 2, entry 10)

Yellow oil; IR νmax (KBr): 3526, 1654, 1614, 1506, 1453, 1396, 810, 759 cm^{-1}; ^{1}H NMR ($CDCl_3$, 400 MHz) δ = 6.89 (s, 1H), 6.87 (d, *J* = 8.0 Hz, 1H), 6.81 (d, *J* = 8.0 Hz, 1H), 5.71 (s, 1H), 3.90 (s, 3H), 3.57 (s, 3H), 3.20 (s, 3H); ^{13}C NMR ($CDCl_3$, 100 MHz) δ = 201.0, 147.0, 145.1, 136.6, 118.5,

112.7, 110.3, 56.0, 44.2, 43.4; HRMS (ESI): calcd for $C_{10}H_{13}NO_2S^+$ [$M+H^+$] 212.0746, found 212.0750.

***N*,*N*-Dimethyl-3-*p*-tolylpropanethioamide (2k, Table 2, entry 11) [5]**

^{1}H NMR ($CDCl_3$, 400 MHz) δ = 7.11 (s, 4H), 3.48 (s, 3H), 3.15 (s, 3H), 3.06 (m, 4H), 2.32 (s, 3H); ^{13}C NMR ($CDCl_3$, 100 MHz) δ = 203.2, 137.5, 135.9, 129.2, 128.3, 45.1, 44.6, 41.5, 35.2, 21.0.

3-(4-Methoxyphenyl)-*N*,*N*-dimethylpropanethioamide (2l, Table 2, entry 12) [5]

^{1}H NMR ($CDCl_3$, 400 MHz) δ = 7.14 (d, *J* = 8.0 Hz, 2H), 6.83 (d, *J* = 8.0 Hz, 2H), 3.79 (s, 3H), 3.47 (s, 3H), 3.14 (m, 4H), 3.05 (s, 4H); ^{13}C NMR ($CDCl_3$, 100 MHz) δ = 203.3, 158.2, 132.6, 129.4, 113.9, 55.2, 45.2, 44.6, 41.5, 34.8.

***N*,*N*-Dimethyl-2-phenylethanethioamide (2i, Table 2, entry 13) [1]**

Obtained starting from acetophenone (**1m**). ^{1}H NMR ($CDCl_3$, 400 MHz) δ = 7.31 (s, 5H), 4.31 (s, 2H), 3.49 (s, 3H), 3.19 (s, 3H); ^{13}C NMR ($CDCl_3$, 100 MHz) δ = 200.6, 135.6, 128.8, 128.3, 128.0, 50.9, 44.8, 42.2.

***N*,*N*-Dimethyl-2-*p*-tolylethanethioamide (2n, Table 2, entry 14) [1]**

^{1}H NMR ($CDCl_3$, 400 MHz) δ = 7.21 (d, *J* = 7.6 Hz, 2H), 7.12 (d, *J* = 7.6 Hz, 2H), 4.26 (s, 2H), 3.48 (s, 3H), 3.20 (s, 3H), 2.35 (s, 3H); ^{13}C NMR ($CDCl_3$, 100 MHz) δ = 200.9, 136.5, 132.6, 129.4, 128.0, 50.5, 44.8, 42.2, 21.0.

***N*,*N*-Dimethylbutanethioamide (2o, Table 2, entry 15) [6]**

^{1}H NMR ($CDCl_3$, 400 MHz) δ = 3.51 (s, 3H), 3.32 (s, 3H), 2.84 (t, *J* = 7.2 Hz, 2H), 1.83 (m, 2H), 1.0 (t, *J* = 7.2 Hz, 3H); ^{13}C NMR ($CDCl_3$, 100 MHz) δ = 204.8, 45.5, 44.7, 41.3, 23.9, 14.1.

***N*,*N*-Dimethylpentanethioamide (2p, Table 2, entry 16) [6]**

^{1}H NMR ($CDCl_3$, 400 MHz) δ = 3.45 (s, 3H), 3.28 (s, 3H), 2.63 (t, *J* = 7.6 Hz, 2H), 1.66 (m, 4H), 0.92 (t, *J* = 7.6 Hz, 3H); ^{13}C NMR ($CDCl_3$, 100 MHz) δ = 205.0, 45.2, 44.5, 41.9, 32.1, 22.7, 14.0.

NMR spectra of new compounds

4-Fluoro-*N*,*N*-dimethylbenzothioamide (2f, Table 2, entry 6)

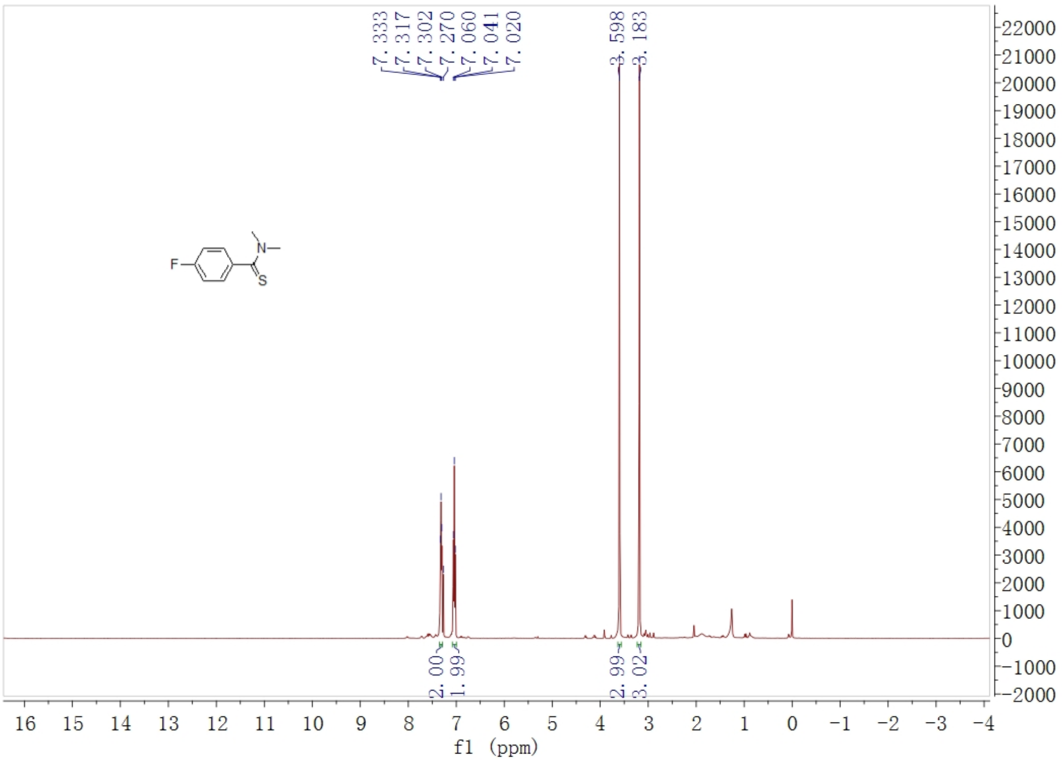

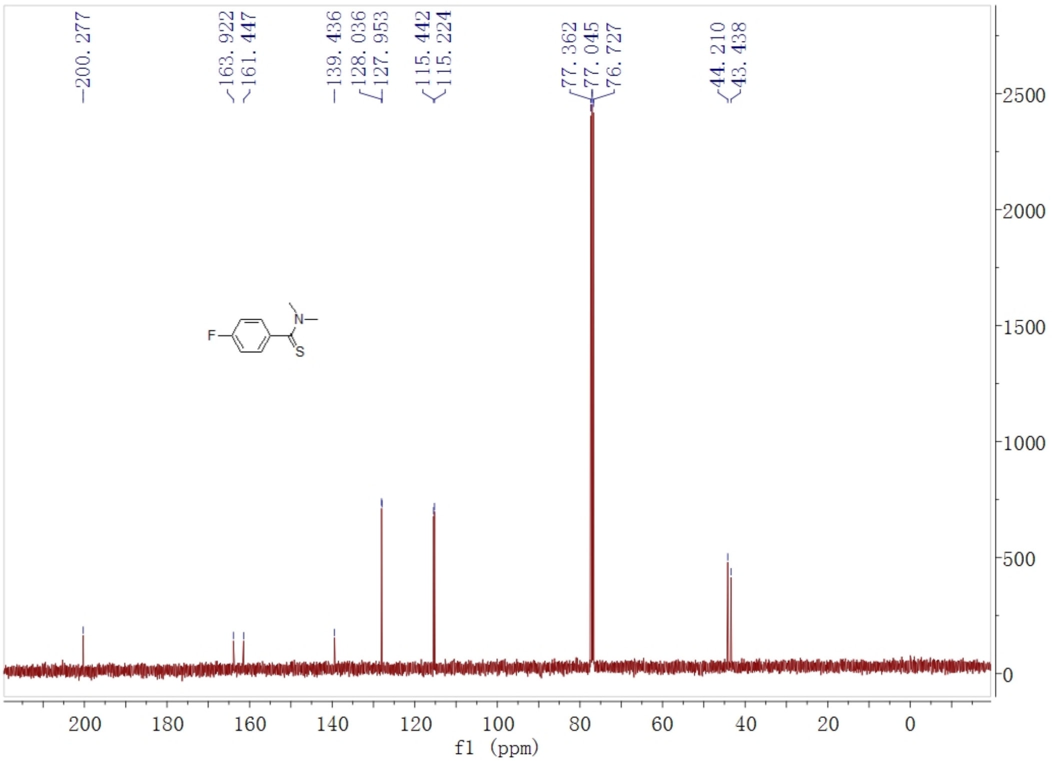

3-Hydroxy-4-methoxy-*N*,*N*-dimethylbenzothioamide(2j, Table 2, entry 10)

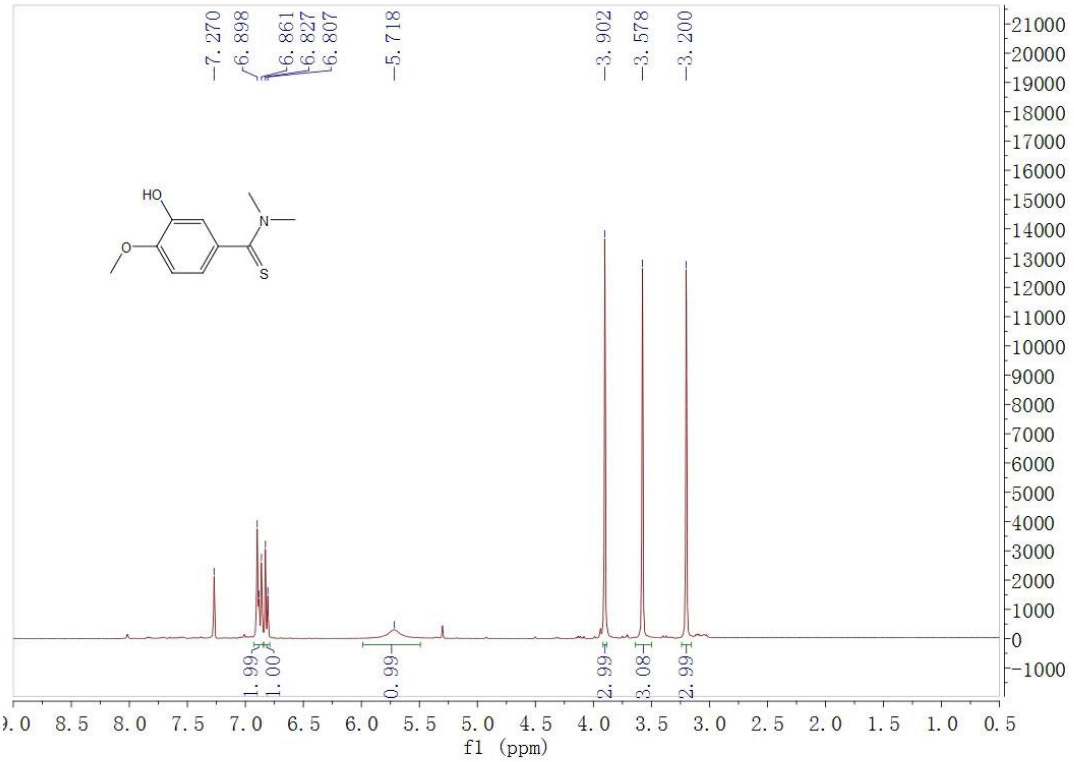

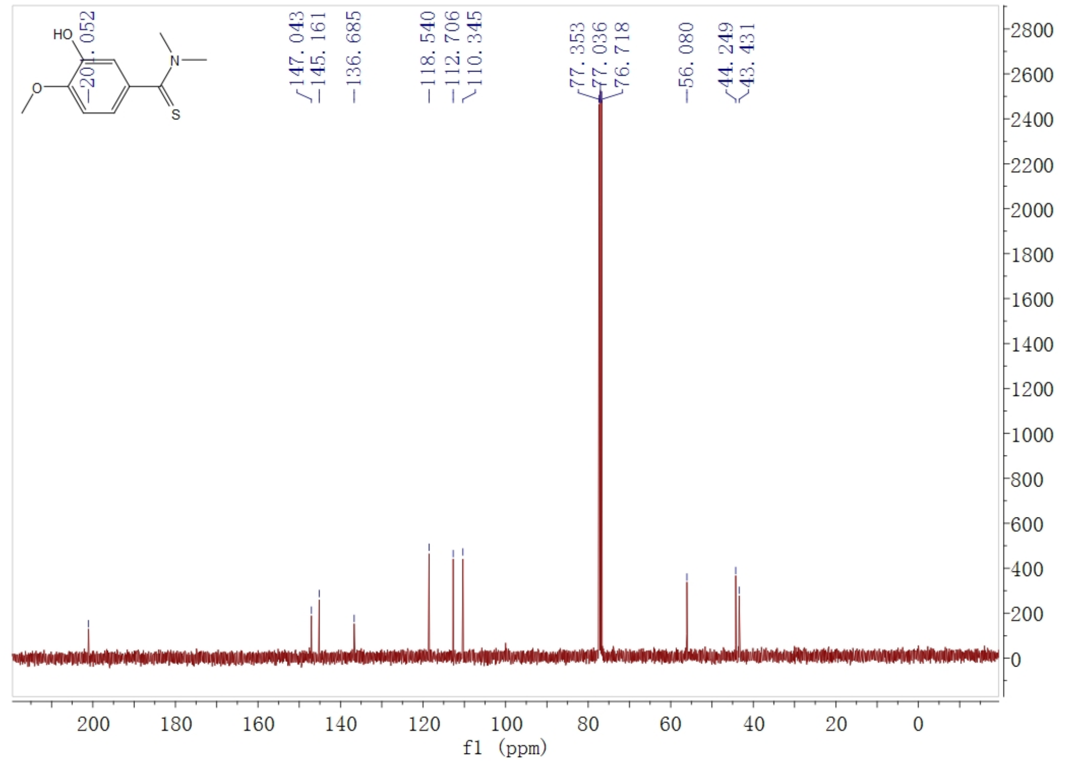

References

[1] Qu, Y. Y.; Li, Z. K.; Xiang, H. F.; Zhou, X. G. *Adv. Synth. Catal.* **2013**, *355*, 3141–3146.

[2] Tirumaleswararao, G.; Rajeshwer, V.; Nand, S. K. *Org. Lett.* **2014**, *16*, 362–43627.

[3] Kaboudin, B.; Malekzadeh, L. *Synlett* **2011**, 2807–2810.

[4] Patrick, B. J.; Merle, T. *J. Chem. Soc., Perkin Trans. 1* **1974**, 863–866.

[5] Karl, K.; Tsauping, L. *Ber. dtsch. chem. Ges.* **1941**, *74B*, 321–327.

[6] Vallée, Y.; Masson, S.; Ripoll, J. L. *Terrahedron* **1990**, *46*, 3921–3928.
